# Does Phage Therapy Need a Pan-Phage?

**DOI:** 10.3390/pathogens13060522

**Published:** 2024-06-20

**Authors:** Petros Bozidis, Eleftheria Markou, Athanasia Gouni, Konstantina Gartzonika

**Affiliations:** 1 Department of Microbiology, Faculty of Medicine, School of Health Sciences, University of Ioannina, 45110 Ioannina, Greece; kgartzon@uoi.gr; 2 Department of Microbiology, University Hospital of Ioannina, 45500 Ioannina, Greece; eleftheria.markou4@gmail.com (E.M.); athgouni@gmail.com (A.G.)

**Keywords:** bacteriophage, phage therapy, host range, broad host range, narrow host range, polyvalent phage, monovalent phage

## Abstract

The emergence of multidrug-resistant bacteria is undoubtedly one of the most serious global health threats. One response to this threat that has been gaining momentum over the past decade is ‘phage therapy’. According to this, lytic bacteriophages are used for the treatment of bacterial infections, either alone or in combination with antimicrobial agents. However, to ensure the efficacy and broad applicability of phage therapy, several challenges must be overcome. These challenges encompass the development of methods and strategies for the host range manipulation and bypass of the resistance mechanisms developed by pathogenic bacteria, as has been the case since the advent of antibiotics. As our knowledge and understanding of the interactions between phages and their hosts evolves, the key issue is to define the host range for each application. In this article, we discuss the factors that affect host range and how this determines the classification of phages into different categories of action. For each host range group, recent representative examples are provided, together with suggestions on how the different groups can be used to combat certain types of bacterial infections. The available methodologies for host range expansion, either through sequential adaptation to a new pathogen or through genetic engineering techniques, are also reviewed.

## 1. Introduction

Many academic institutions and private businesses have turned to phage therapy in response to the sharp rise in the appearance and dissemination of antibiotic-resistant pathogens over the last decades, as well as the dearth of novel drug development [1,2,3]. The term “phage therapy” refers to the use of lytic bacterial viruses, bacteriophages (literally meaning “bacteria eaters”), which are capable of infecting, lysing, and eventually eliminating harmful bacteria [4,5]. Although there were hints in the literature even before Frederick Twort’s publication in 1915 on “the nature of ultra-microscopic viruses”, it was Felix D’Herelle who had the idea that phages could be used as biocontrol agents [6,7,8]. Since then, the application of phages has shown enormous potential in human and veterinary medicine, agriculture, and the food industry [9,10,11]. With respect to human medicine, phages were used for the treatment of several conditions, including dysentery, cholera, and staphylococcal skin diseases, almost immediately after their discovery [12,13,14]. At a time when options for bacterial disease treatment were limited, the enthusiasm for this new effective tool was so great that even commercialized phage products for human use appeared on the market by companies such as Eli Lilly during the 1930s [15]. However, despite the confirmation of the particulate nature of phages through electron microscopy in 1940 and increasing comprehension of phage biology, interest in phage therapy started to fade because of the discovery and clinical use of antibiotics [16,17].

The rise and prevalence of antibiotics in the treatment of bacterial infections were based on key advantages that they had. These include their effectiveness against various infectious diseases, ease of use, and relatively low cost of production, as well as the stability of their preparation process [18]. The main advantage of antibiotics, however, was their broad spectrum of activity. Streptomycin, for example, one of the first aminoglycoside antibiotics, was discovered in 1943 and used to treat tuberculosis [19]. In addition to that, it was also approved for broad clinical use in 1946 because it exhibited antimicrobial activity against multiple Gram-positive and Gram-negative bacteria, in addition to *Mycobacterium tuberculosis* [20,21]. A plethora of other antibiotics, like chlortetracycline or trimethoprim, have found their way as clinical agents for the treatment of bacterial infections caused by both Gram-positive and Gram-negative bacteria [22,23,24]. In contrast, bacteriophages are known to be specific to a particular species or strain of bacteria. This ability of bacteriophages to specifically infect and lyse their host has been acknowledged since the early days of phage discovery and utilized for the characterization and epidemiological identification of different strains within a given species through a procedure known as “phage typing” [25].

Host range is critical for phage therapy. Despite the description of seven types of host range, such as adsorptive, penetrative, bactericidal, productive, plaquing, spotting, and lysogenic, each representing discrete infection steps that a phage can successfully complete, the productive types, particularly the plaquing ones, are the key determinants for phage therapy [26]. The productive type refers to the species or strain of host bacteria on which a phage is able to establish an infection that produces progeny that is released after cell lysis (as far as lytic phages are concerned). The productive host type may also be a plaquing type if the phage is able to form plaques on the host lawn, or it may not be a plaquing type in the case of a host that allows only a limited number of progenies [27]. Within the context of phage therapy, the host range of a lytic bacteriophage could be seen as a two-sided coin: on the one hand, the use of a strictly specific phage will not affect the non-pathogenic bacteria during treatment, while on the other hand, this same specificity restricts the use of a given phage to a limited range of potential pathogens [28,29]. A suggested method for addressing phage specificity was to use what is known as a “phage cocktail” [30]. Phage cocktails can include a combination of narrow-range phages that aim to kill either different pathogenic bacterial species or different strains of a particular species. Since many lytic phages can be mixed to create a far wider lytic spectrum, the concept of a phage cocktail allows the employment of lytic phages with limited host ranges. Multivalent phage cocktails can be useful against a variety of pathogenic strains of the host, and because they contain a variety of phage components, they can slow down the emergence of resistance significantly [31,32]. In addition, the effectiveness of the phages in the cocktail has been reported to be enhanced, albeit not always, when compared to each of the phages individually [33,34]. The alternative path for phage therapy is “monophage therapy”, in which a single phage, usually with a broad host range, is used. Both monophage therapy (or monotherapy) and phage cocktails were used in one of the two main phage therapy approaches, which is known as personalized or customized treatment [35]. Personalized treatment is essentially precision medicine tailored to the individual patient. In this approach, single phages or targeted phage cocktails are employed directly, depending on the pathogen isolated from the patient. This method is therefore more suitable in terms of the phage host range, effectively reduces the emergence of bacterial resistance through adaptation (see below), and has better outcomes for the patient [36]; however, it must be supported by local, well-characterized phage banks, which are phage collections that are able to sustain a large number of clinical isolates of a given bacteria strain [37]. These collections also need to be renewed and enriched from time to time. The second approach to phage therapy involves the use of so-called “fixed cocktails”, in which many lytic phages are selected against one or more bacterial species as long as their combined action is able to broaden the overall host range of the mixture [30]. These predefined formulations against the most common Gram-positive and Gram-negative pathogens have been developed and applied, with the idea that phage therapy could be a direct alternative to the inadequacy of antibiotics that act against a wide range of bacteria.

The terminology describing the host range often suffers from confusion as the same terms are used to describe different phenomena. For example, the term “broad host range” is used both to describe phages that are able to infect multiple strains of a single species as well as phages that are able to infect multiple species within a genus or even several genera [38,39]. In the end, the host range is the result of a sum of extracellular and intracellular restrictions that are applied through the process of infection. Collectively, all these restriction mechanisms consist of what is known as the “bacteriophage resistome”. The resistome includes mechanisms such as adsorption resistance, phage-genome uptake block, superinfection immunity, restriction enzymes, anti-phage defenses like CRISPR, and abortive infection; yet, the limiting factor that defines the potency for infection is the presence of the appropriate receptor and its availability to the phage [26,40].

## 2. Host Range Determination Starts with the Initiation of Infection

### 2.1. Cell Surface Receptors of Bacteriophages

Certain strains of bacteriophages exhibit strict host specificity, targeting specific microbial strains or species. This specificity is primarily driven by the adsorption process, which hinges on the structural features and types of receptors present on the bacterial surface [41]. Recent advances in understanding the structural organization of tail fibers, such as those of the T4 phage, highlight how the structural–functional relationships of these fibers play a crucial role in recognizing host surface receptors, suggesting potential strategies for reprogramming phage host ranges in order to improve the effectiveness of phage therapy [42]. The distribution and density of these receptors on the cell surface are also critical, influencing how bacteriophages interact with different taxonomic groups. These interactions are generally dictated by the composition and characteristics of the host cell wall.

### 2.2. Receptors Located in the Cell Walls of Gram-Positive Bacteria

The cell walls of Gram-positive bacteria are rich in peptidoglycan, which constitutes 40–90% of the cell’s dry weight. This complex polymer, composed of N-acetylglucosamine and N-acetylmuramic acid units, is not only crucial for maintaining cellular integrity but also serves as a binding site for other molecules. Unlike Gram-negative bacteria, the peptidoglycan layer of Gram-positive bacteria is exposed on the cell surface, making it accessible for phage attachment [43]. Phages infecting Gram-positive bacteria often use a carbohydrate component on the host cell surface as a receptor, such as cell wall polysaccharides for phages 936 and p335 infecting *Lactococcus lactis* [44]. In the case of *Clostridium botulinum* phage a2, glycan moieties of the cell wall peptidoglycan were suggested to be involved in the receptor site of the phage [45].

In Gram-positive bacteria, surface proteins are linked to peptidoglycan either by ionic interactions or by covalent bonds. Such proteins are used as phage receptors, although only a limited number have been discovered among Gram-positive strains; for example, *Bacillus anthracis* phage γ binds to GamR, a cell wall-anchored protein [46]. On the other hand, integral membrane proteins also serve as binding sites for phages. This has been demonstrated for the integral proteins PIP, PIPEF, and YueB for *Lactococcus lactis* phage c2, *Enterococcus faecalis* phages (φVPE25 and φVFW), and *Bacillus subtilis* phage SPP1, respectively [47,48,49]. Gram-positive cell walls contain large quantities of wall teichoic acids [50], accounting for approximately 60% of their dry weight [51]. Gram-positive bacteriophages often attack these wall teichoic acids as their quantity makes them ideal targets [50]. A Gram-positive bacterium virus called phage 3C is an example of a phage that specifically binds to the N-acetyl glucosamine moiety of teichoic acids on the surface of *Staphylococcus aureus* [52]. Another example is the D-glucose chain of the teichoic acid located on the surface of *Bacillus subtilis*. This chain serves as the receptor for phages SP2 and SP10 [53]. Lipoteichoic acids (LTAs) can also be used for phage binding, as exemplified by phage LL-H, which specifically targets *Lactobacillus delbrueckii* bacteria via LTAs [54]. Initially, the phage receptor binding protein binds reversibly to the glucose moiety at the surface end of the LTA via one of its C-terminal domains, and later, a second domain provides irreversible binding to the glycerol phosphate group near the surface end of the LTA [54].

### 2.3. Receptors Located in the Cell Walls of Gram-Negative Bacteria

Receptors in the cell walls of Gram-negative bacteria have a distinct configuration, markedly differing from those in Gram-positive bacteria. A notable feature of Gram-negative bacteria is the high permeability of their outer membrane, attributed to an abundance of integral proteins that form transport channels [55]. This outer membrane contains a lipopolysaccharide (LPS), a compound typically unique to Gram-negative organisms, playing a crucial role in phage attachment [56]. The outer membrane’s lipid bilayer comprises an inner phospholipid layer and an outer glycolipid layer dominated by an LPS. The LPS itself consists of lipid A, a core polysaccharide, and an O-antigen, serving as primary receptors for phages targeting Gram-negative hosts [57,58]. The *Escherichia coli* phage T3 and U3 require the glucosyl alpha-1,3-glucose terminus of a rough LPS and the terminal galactose residues in an LPS to initiate adsorption, respectively [58,59]. Phages interact with either the variable O-antigen or the more stable core polysaccharide. For example, *E. coli* phage T5, which first reversibly binds to O-antigen and then irreversibly attaches to the FhuA protein localized in the cellular outer membrane, displays high specificity, whereas those that bind to the core polysaccharide, such as the *Yersinia pestis* phage PST, typically have a broader host range [53]. The *Pseudomonas aeruginosa* phages E79 and JG004 both require the core polysaccharides of the LPS to adsorb to their host [60,61]. In addition to the LPS constituents alone, there are numerous phages that require a co-receptor, in which the LPS structure may serve as the secondary receptor where irreversible binding occurs. A prominent example is the *E. coli* K-antigen serotypes, where phages targeting them require the respective outer polysaccharide capsule as the primary receptor. Phage PNJ1809-36 requires both the K1 capsule and the LPS to initiate infection [62].

Proteins, another integral component of the outer membrane, form a physical barrier but also facilitate transport via porins such as OmpF and OmpC [63]. These proteins allow the passage of small molecules and are commonly identified as phage receptors in Gram-negative bacteria. The *Salmonella* phage S16 requires OmpC in addition to the LPS outer core to fully adsorb to the host [64]. Another *Salmonella* phage, ES18, requires the β-barrel and cork domains of the outer membrane protein FhuA, which is required in ferrochrome transport [65]. In addition, TolC is known to be used as a receptor for phages infecting *Salmonella enterica* serovar Typhimurium [66].

### 2.4. Receptors in Additional Structures of Gram-Positive and Gram-Negative Bacteria

The S-layer, forming the outermost envelope of certain bacterial and archaeal cells, is composed of proteins or glycoproteins arranged into a two-dimensional crystalline lattice. This structure not only plays a pivotal role in maintaining cell surface integrity through noncovalent interactions with components such as the LPS or secondary cell wall polysaccharides but also features pores ranging in size from 2 to 10 nanometers [67]. These pores are critical in mediating interactions with the environment, including the initial attachment of bacteriophages, which can be crucial for phage infectivity. The S-layer has been shown to be a receptor for at least *Bacillus anthracis* phage AP50 [68], *Caulobacter crescentus* siphovirus phiCr30 [69], and *Lactobacillus helveticus* myovirus CNRZ 832-B1 [70].

A capsule composed primarily of polysaccharides secured to peptidoglycan may envelop a cell wall as another protective structure. The main function of a capsule is to ward off phagocytosis and desiccation while aiding bacterial adhesion and pathogenicity and concealing receptor sites located on cell walls [40]. However, some phages have the ability to attach themselves specifically to capsules found on *E. coli*, *Klebsiella pneumoniae*, *S. enterica*, and *Acinetobacter baumannii* [71,72,73,74]. Bacteria have the capability of synthesizing exopolysaccharides, an unusual type of polysaccharide. Unlike capsular polysaccharides, which strongly bind to cell walls (known as the slime layer), exopolysaccharides are either directly released into the extracellular environment or weakly attached via the slime layer [75,76]. One study demonstrated that exopolysaccharide serves as the main receptor for *Enterococcus faecalis* phage NPV1 [77].

Flagella and pili are appendages located on the surfaces of both Gram-negative and Gram-positive bacteria. Flagella facilitate bacterial movement, while pili play roles in both adhesion to cells and surfaces and genetic exchange between Gram-negative bacteria. *Caulobacter crescentus* phages Cb13 and CbK interact with the flagella using a head filament, followed by an irreversible attachment to a surface receptor via the phage tail fibers [78]. Several phages that infect *Pseudomonas* spp. specifically recognize and bind to type IV pili. It is believed that the retraction of these pili brings the phages into closer proximity to the bacterial surface, allowing them to establish irreversible connections with their specific receptors [79,80]. Conjugative pili are often used as receptors for plasmid-dependent bacteriophages (PDPs), which target these plasmid-encoded secretory structures on the bacterial cell surface [81,82]. PDPs are dependent on specific plasmids and can only infect plasmid-containing bacteria. One of the best-studied PDPs is phage PRD1, which is dependent on the incompatibility group P, W, or N conjugative plasmid and can infect a variety of Gram-negative hosts, including *Salmonella* Typhimurium, *E. coli*, and *Pseudomonas putida*, provided that they harbor the appropriate plasmid [82,83].

Mycobacteria, such as *M. tuberculosis*, have a distinctive cell wall composition. These microorganisms are classified as Gram-positive bacteria, but they possess extra layers in their cell wall that share some characteristics with the outer membrane [84]. Most of the *M. tuberculosis* cell wall is composed of peptidoglycan, arabinogalactan, and mycolic acid, which are chemically linked together. This membrane also contains proteins that have a structural arrangement like that of porins in Gram-negative bacteria [85]. Based on the existing evidence, the lipid molecules of the cell wall are often identified as the phage receptors of mycobacteria. The *Mycobacterium smegmatis* phage I3 utilizes glycopeptolipid molecules as receptors, which consist of a tetrapeptide covalently attached to lipid moieties and a methyl-rhamnose residue [86]. However, receptors have not yet been identified for the overwhelming majority of mycobacteriophages.

Although it is widely believed that no phages infecting both Gram-positive and Gram-negative bacteria have been detected, there are rare reports of such phages in the literature [87,88]. However, in these reports, the results are controversial and have not been further confirmed. It therefore remains to be proven that such phages capable of infecting both Gram-negative and Gram-positive bacteria truly exist.

## 3. How Broad the Host Range Can Be

Several metagenomic studies conducted on environmental samples have suggested the potential wide distribution of broad-host-range phages in nature [89,90]. As previously mentioned, the literature uses the term “broad” to describe various phenomena. To fully understand a phage’s potential in phage therapy, it is generally best to classify newly isolated and characterized phages based on the taxonomic level of the host range they span. We have collected indicative examples of phages reported in the literature during the last five years and classified them into three categories (Host Range Classes I, II, and III) depending on the taxonomic rank of the hosts. We propose this classification to be established for clarification purposes. The examples are displayed in Table 1.

## 4. Human Tract Infections from the Host Range’s Point of View

One of the major advantages of phage therapy is considered to be the selective eradication of target bacteria, without affecting the host microbiota. This characteristic, along with its compatibility with antibiotics and low immunogenicity, has drawn clinical interest in treating bacterial infections caused by multidrug-resistant bacteria [5]. Below, we discuss the potential of phage therapy in the context of the applied host range for three crucial categories of bacterial infections.

### 4.1. Urinary Tract Infections (UTIs)

Among the most common community and nosocomial infections are urinary tract infections [110]. Both Gram-negative and Gram-positive bacteria may be the cause of UTIs, including *E. coli* (80%), *Staphylococcus saprophyticus*, *P. aeruginosa*, *K. pneumoniae*, *Enterobacter* spp., *Enterococcus* spp., group B *Streptococcus* (GBS), *Proteus mirabilis*, and *S. aureus* [111,112]. Several attempts to explore the efficacy of phage therapy in urinary tract infection treatment have been reported during the past few years [113,114]. Monotherapy, or a cocktail of phages were both used, revealing that either single multivalent phages or a cocktail of broad-range phages may overcome the phage resistance mechanisms raised by uropathogenic strains [115,116,117,118].

In regard to UTI treatment by phage therapy, it seems that the decision on the host range of the applied lytic phages should be based on the following facts:

(a) There was a long-standing belief that the bladder and urethra of healthy individuals are devoid of bacteria or contain insufficient numbers to cause infections; however, recent reports have demonstrated the presence of numerous microorganisms in the bladder of healthy adults without clinical manifestations [119,120]. Further research is required to determine the significance and contribution of these microorganisms to health and disease [119,120].
(b) UTIs are frequently caused by bacterial biofilms, which account for approximately 65% of nosocomial infections and 80% of all microbial infections [121]. Diverse bacteria disperse biofilms via a variety of mechanisms during their transmission [122]. Biofilms are also found within host cells, where they establish intracellular bacterial communities (IBCs) that serve as a protective barrier against neutrophils and antibiotics, thereby significantly contributing to the progression of recurrent urinary tract infections [123]. Specifically, in cases of catheter-associated UTIs (CAUTIs), which comprise 40% of all hospital-acquired infections, bacterial biofilms have a substantial effect on the development of UTIs [124]. Urothelium, prostate stones, and implanted biomedical devices are all potential sites for biofilm formation caused by both Gram-positive and Gram-negative bacteria [125]. In the early stages of CAUTIs, biofilms are often colonized by a single species; this is followed by the development of mixed communities, leading to the formation of a thick biofilm that renders antibiotic therapy ineffective [125].

Combining all the above data, one could conclude that the design of phage therapy treatment for UTIs may allow for the application of phages that show the greatest possible breadth on the host range scale, as long as this does not disturb the commensal bacterial communities about which little is known. Five clinical trials on urinary tract infections have been carried out in the US since 2022, of which some are ongoing [126]. In one trial that has been completed, the Eliava Institute’s commercially available cocktail (Pyo phage) against eight pathogens (*S. aureus*, *S. salivarius*, *S. pyogenes*, *S. sanguis*, *S. agalactiae*, *E. coli*, *P. aeruginosa*, *P. mirabilis*, and *P. vulgaris*) was used and evaluated in UTI patients following transurethral resection of the prostate. The other two clinical trials employed a CRISPR-Cas3-enhanced phage cocktail against *E. coli* (LBP-EC01), while the final two trials evaluated the safety and efficacy of phage treatments against both *E. coli* and *K. pneumoniae* [126].

### 4.2. Respiratory Tract Infections

Respiratory tract infections are divided into upper respiratory tract infections (URTIs) and lower respiratory tract infections (LRTIs). LRTIs were responsible for 4 million deaths globally in 2019 [127]. *Streptococcus pneumoniae*, *Haemophilus influenzae*, *P. aeruginosa*, *Chlamydophila pneumoniae*, *Legionella pneumophila*, *K. pneumoniae*, *Moraxella catarrhalis*, and *S. aureus* are among the most common causes of bacterial respiratory infections [127,128]. From an early age, a multi-kingdom microbial ecosystem, referred to as the RT microbiome (RTM), colonizes and gradually establishes itself on the respiratory mucous layers in such a way that the diversity, complexity, and quantity of the inhabited taxa decrease from the upper to the lower respiratory tract [129,130]. Among the most abundant phyla (*Firmicutes*, *Bacteroidetes*, *Proteobacteria*, *Actinobacteria*, and *Fusobacteria*) across the RT, the genera *Dolosigranulum* and *Corynebacterium* are considered to play a protective role against infections, whereas microbiomes rich in *Steptococcus*, *Neisseria*, *Haemophilus*, or *Prevotella* are associated with dysbiotic profiles, which may lead to respiratory infections and asthma [131,132]. This becomes more apparent when antibiotics are administered. Although the impact of antimicrobial therapies on the RTM has received less attention, recent data show that exposure to antimicrobial agents disrupts the microbial ecosystem, decreases its diversity, eradicates protective and beneficial taxa, and favors resistant or opportunistic pathogens [133,134].

Accordingly, phage therapy as an alternative to antibiotic treatment for bacterial infections of the lungs and associated tissues was reported almost 60 years ago [135,136]. All the possible schemes for phage application, including phage banks and monophage therapy with single monovalent or polyvalent monophages, phage cocktails targeting either multiple species (or even genera) or a single pathogen species, and combinations of phage therapy with conventional antibiotics, have been used in several respiratory infections [137,138,139,140]. Regardless of the type of phage formulation, the method of administration, or the use of phages only for prophylactic treatment, the common ground of most of these efforts in the treatment of human and animal respiratory infections was the lack of acute or chronic toxicity, the absence or low stimulation of the inflammatory response, and the lack of adverse effects for the patient. In addition, there are several studies that show that phage treatment may also protect the lungs of treated animals from alveolar wall thickening and neutrophil infiltration compared to untreated controls [141]. Also, compared to antibiotics, it has been reported that phage therapy does not give rise to any of the side effects observed when antibiotics are used, such as dybacteriosis and allergic reactions [137]. In the majority of these studies, species-specific phages were used for administration, while polyvalent phages have been used with success, mainly in the former Soviet Union. Because polyvalent phages could be useful in chronic pulmonary infections like cystic fibrosis, which are often polymicrobial, an increasing number of research groups and private companies are investigating ways to broaden the range of hosts, reduce resistance to phages, and enhance the breakdown of biofilms [142]. A good paradigm is the “Adaptive Phage Therapy Protocol” that was created and applied by scientists of the Research and Production Centre (RPC) “Micromir”, in collaboration with clinicians of intensive care units (ICUs) of the Federal Research and Clinical Center of Intensive Care Medicine and Rehabilitology (FRCC ICMR), for the prevention and treatment of all ICU patients [143]. The protocol is based on the selection of clinical isolates every 30 days, which then are examined by RPC personnel for their sensitivity in the phages in its phage bank. After the examination, the RPC propagates an adapted (see below) phage cocktail with an efficacy of at least 70% against each species of isolated bacteria and transfers the adapted cocktail to the FRCC ICMR in order to be applied to the ICU patients two to three times per day. The difference compared to the other therapeutic protocols lies is that the technology of the therapy implies the strict compliance of a set of bacteriophages to the needs of a particular ICU rather than a particular patient [143].

Although the use of expanded-host-range phages may provide treatment solutions, very little is known about their effect on RTM. In fact, little is known about the commensal bacteriophages of the respiratory tract regarding their role in the community’s ecology and their interaction with the host. As far as DNA bacteriophages are concerned, it is believed that a resident core group of 19 phages is present in the human respiratory tract, which directly influences the bacterial network in health and disease, whereas a large portion of the virome sequences remain unclassified [144,145]. A study examining the respiratory virome and serum cytokine profile in cases of recurrent acute respiratory tract infections (ARTIs) in children found an association between the high frequency of *Propionibacterium* phages/low frequency of *Lactococcus* phages and multiple ARTIs, as well as excessive airway inflammation induced either by bacterial pathogens or bacteriophages themselves [146]. As we decipher the intricacies of the RTM ecology and its underlying mechanisms in health and disease, several formulation types of variable-host-range phages in prophylaxis-assisted, restoration-assisted, and treatment-assisted strategies are expected to be used, along with other microbiome-based therapies.

### 4.3. Gastrointestinal Tract Infections

Gastrointestinal tract infections (GTIs) account for around 5 billion cases worldwide and are responsible for 1.4 million deaths every year, with a notable contribution to infant mortality [147,148]. Some of the most common bacterial pathogens causing outbreaks of GTIs are *E. coli*, *S.* Enteritidis, *Campylobacter jejuni*, *Listeria monocytogenes*, *Clostridium difficile*, and *Vibrio cholerae*.

Phage therapy for the treatment of gastrointestinal diseases has been used for more than a hundred years and has led to promising treatment solutions for infectious diseases, such as bacterial dysentery and cholera, before the discovery of antibiotics [149]. Since then, phage therapy has been applied in several formulations for GTI treatment, especially in the former Soviet Union, although there is not enough available information on the host range of the phages used [150]. Following the recent rise in popularity of phage therapy in the West, the treatment used in pre-clinical studies on animals and the few reported cases of phage therapy in humans were either monotherapy (using a phage that is specific to a single species) or phage cocktails, which were made up of multiple species-specific phages that made the mixture more effective against a wider range of hosts within the same species [151,152,153]. Researchers have also tested both approaches in combination with antibiotics. Most animal studies showed that phage therapy significantly reduced enteric pathogens several hours after infection, delaying or preventing disease onset, whereas prophylactic applications appeared to be less effective.

All these efforts, reflecting the transition of phage biology from experimental to clinical settings, have gradually taken into account the accumulating information on host targets and their behavior in their natural environment, i.e., the gut. Designing and applying phage therapy within the framework of the human gut microbiome, the most extensively studied microbiome in our body, are therefore crucial. During the past decade, there has been a burst of information about the gut microbiota, which consists of trillions of microorganisms that reside in the gastrointestinal tract and have a leading role in health and disease [154]. Despite the primary focus on bacterial group characterization, researchers have also gathered extensive information on the virome’s composition, particularly the phageome [155]. We now know that the most common dsDNA phages in the gut are those belonging to the *Siphoviridae*, *Myoviridae*, *Podoviridae*, *Corticoviridae*, and *Tectiviridae* families, as well as ssDNA phages belonging to the *Microviridae* and *Inoviridae* families, whereas a large proportion of phages (>50%) remain unclassified or unknown [156]. Interestingly, groups such as crAssphages remain conserved and found across unrelated individuals, despite the high variability in phage communities among the populations examined so far. Most of these phages are temperate and are incorporated into the bacterial chromosomes as prophages [157]. Every organ and tissue of the GI tract contains phages, which can directly influence the numbers and functions of host bacteria, thereby defining the microecological balance and imbalance, indirectly contributing to gut microbiome-associated diseases [158]. In the past ten years, 16S rRNA sequencing by NGS has revealed that *Bacteriodetes*, *Firmicutes*, *Actinobacteria*, *Proteobacteria*, and *Verrumicrobia* are the most common phyla in the digestive tract, while *Enterococcus*, *Coprobacillus*, *Escherichia*, and *Shigella* are the most common genera [159]. Not surprisingly, the composition of the gut microbiota of healthy individuals differs from that of gastrointestinal disease patients. Several factors, such as stress, poor nutrition, inflammatory and chronic conditions, the use of antibiotics or drugs, and surgical procedures, can alter the number and diversity of the intestinal microbiota, leading to a condition known as “dysbiosis” [154]. Dysbiosis can lead to unfavorable outcomes, including gastrointestinal disorders, metabolic disorders, autoimmune disorders, and other diseases.

**Figure 1:**
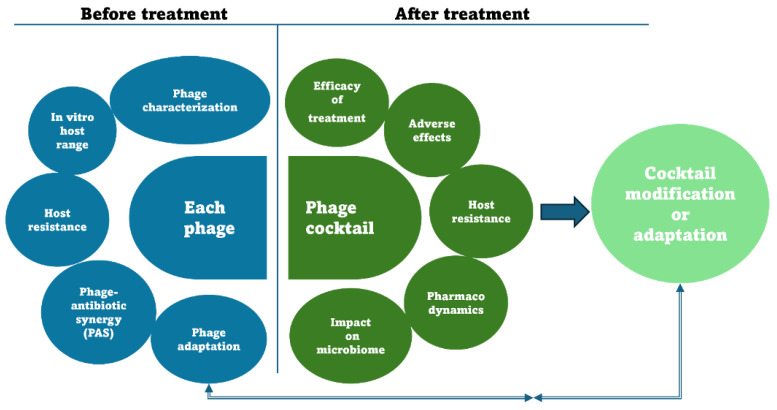
Basic levels of phage therapy evaluation before and after treatment. The characterization of the phage necessarily includes the characterization at the genomic level, whether it is a phage isolated from the environment or a phage obtained from a bank. Phage-antibiotic synergy refers to the synergistic interaction between antibiotics and phages [171]. Ideally, the future post-treatment evaluation of phage therapy should integrate the impact of treatment on the microbiome through appropriate metagenomic analysis.

Phage therapy has already been tested for providing solutions against such dysbiotic conditions [152]. Successful attempts with such treatments have demonstrated that the use of phage cocktails and multiple treatments resulted in better results [153]. Most importantly, the in vitro specificity and efficacy of phage cocktails should be tested against both pathogenic strains that promote dysbiosis and non-pathogenic strains of the same species associated with a healthy microbiome [160]. After phage application, not only should the activation of inflammatory responses be monitored but metagenomic data should also be collected and evaluated to ensure that the long-term administration of the therapy does not itself lead to dysbiosis [161]. Metagenomic data analysis, however, should examine both the composition of the commensal bacteriome and the phageome. There is a plethora of data that correlate alterations of the phageome with human diseases, just as alterations of the bacteriome do [162,163]. Until now, metagenomic analyses have focused exclusively on changes in the bacteriome. However, most of its members harbor lysogenic phages, often more than one (polylysogeny) [164]. Different stimuli, such as antibiotics, can induce phages in a lysogenic state to enter the lytic cycle, which is both dependent on the SOS response and independent of it [165,166]. Such a shift from predominantly temperate to virulent phages may lead to dysbiosis. Relevant studies suggest that a shift from the lysogenic to the lytic cycle in the gut could lead to inflammatory bowel disease (IBD) [167]. Meanwhile, Microbiota Transfer Therapy treatment in the gut of autism spectrum disorder patients restored phage composition and increased bacterial diversity, indicating phages’ potential contribution to gut dysbiosis [168]. The effect of phage therapy on the potential activation of the gut flora’s lysogenic phages is still unknown. There is no evidence to exclude the horizontal exchange of genetic material between therapeutic phages or non-therapeutic phages, which are present as prophages in the bacterial population or as phages that act in situ during multiple rounds of infection and lysis in a dense and relatively localized bacterial population [169]. As a result, future metagenomic analyses should also be able to evaluate alterations in phage composition. The problem is that appropriate bioinformatics tools to distinguish virulent and temperate phages are not yet available. To that end, the development of computational tools, such as DeePhage and other notable works, promises a more complete characterization of several phage-mediated microbiome treatments [170]. In conclusion, the introduction of foreign prokaryotic viruses through phage treatment could, in ideal conditions, make use of the procedures described in Figure 1.

## 5. Broad-Range Phages Should Be Either Made by Expansion or by Synthetic Biology

The implementation and establishment of phage therapy as a contemporary and effective means of antimicrobial therapy requires methods of the quick and easy development of a new generation of therapeutic phages. The choices are concrete: (1) we will have to develop techniques of selection and isolation of broad-range phages from the environment; (2) we will need to apply and evolve techniques of expanding the host range of existing phages, or (3) we must develop genetic engineering techniques to manufacture such kinds of phages. The last two alternatives will be discussed below.

### 5.1. (A) Methods for the Experimental Expansion of the Host Range

Phage adaptation, or phage training, refers to the experimental ways of exploiting the phage’s natural capability to evolve, overcome bacterial resistance, and infect a non-permissive bacterial host. The first such attempt comes from the early 1920s and originates from the Appelmans experiment [172]. Appelmans developed the principle that a population of phage progeny is not uniform but includes several variants that have different characteristics from the rest, e.g., resistance to toxic chemicals and, by extension, the ability to infect a new host. Appelmans‘ idea was to distinguish these phage variants through serial phage dilutions, in which phages are challenged against the selection factor and variants are selected. Based on Appelmans’ principle, subsequent phage training experiments typically involved either overnight co-incubation of a specific concentration of bacteria with serial dilutions of the phage or longer periods of co-incubation of the phage and bacteria [173]. Usually, the successful adaptation appears after several “passages” in both approaches.

#### 5.1.1. Single Phage Adaptation to a New Host (Examples of Host Range Expansion within Species)

Laanto and co-workers studied the ability of the myophage FCV-1 to overcome the developed host resistance of *Flavobacterium columnare* strain B245, which is the phage’s natural host. The researchers co-cultured the host and the phage for a four-week period in lake water without nutrient additions at MOI 1. After one week of incubation, they observed an increase in the phage-resistant rough morphotype of *F. columnare*, which suggests that the host altered the surface structures as a primary defense mechanism. Phage evolution produced infectious phages capable of infecting all types of bacteria from day 7 of the incubation, while sequencing revealed that this ability may be due to mutations in putative tail proteins. However, the cost for this adaptation was the lower adsorption of the evolved phage [174]. In another coevolution study, Borin and colleagues incubated populations of *E. coli* B strain REL606 with λ phages in flasks for 30 days at MOI 10^4^ or 10^3^, which were either untrained lytic strains that use LamB as a receptor or trained lytic strains that may use two receptors for infection, namely, LamB or OmpF. In comparison to the treatment with untrained phages, the researchers found that treatment with trained phages led to improved and prolonged suppression because it was more difficult for bacteria to evolve resistance to trained phages. The reason for the latter is that since trained phages target two receptors instead of one, bacteria should acquire complete resistance through multiple mutations, which in turn, may impose a fitness cost on the bacteria. The researchers concluded that although the role of phage adaptation in the efficacy of in vivo therapy has yet to be demonstrated, the ability of trained phages to coevolve with and counter host defenses may improve treatment outcomes [175]. Similarly, Luzon-Hidalgo and colleagues recently published an analytical protocol for the study of phage adaptation to new hosts [176]. One of the biggest problems of phage therapy is that during treatment, phage-resistant isolates (“resisters”) arise. In a mouse model, Salazar and colleagues demonstrated that this is also the case following treatment with the φHP3 phage of the pandemic *E. coli* strain ST131 infection. The resisters that were developed harbored mutations in genes encoding either the system that synthesizes LPS or OmpA. The researchers showed that this resistance was developed at a cost since the resisters were forced to exchange fitness in other environments of the body, like blood. Moreover, they showed that the phage φHP3 could be trained against the resisters again to give a new mutant phage that could efficiently lyse all the resisters, as well as their parents. As a novel procedure in this study, an automated bacterium–phage bioreactor that continuously cycles fresh phages grown on its original bacterial host (parental strains) into a chamber that contains the target bacterium (resistant isolates) was used to facilitate the phage–bacterial arms race [177]. In another coevolution study of *E. faecium* strain TX1330 with the well-characterized broad-range phage EfV12-phi1, which has the potential to lyse species of *Enterococcus*, *Streptococcus*, and *Staphylococcus*, the phage was grown with *E. faecium* with 1:10 serial transfers twice daily for 8 days at an MOI of 0.003. These experiments showed that coevolved bacterial isolates developed resistance to ancestral phages through exopolysaccharide and RNA polymerase mutations. As a counter mechanism, the phages evolved through a previously unknown phage escape strategy involving large tandem repeats in the tail fiber gene, which broadened their range, and thus, they were able to infect both the ancestral bacteria and the resisters [178].

#### 5.1.2. Generating Phage Cocktails of Better Efficacy through Experimental Adaptation

Although undesired outcomes (e.g., decrease in host range and loss of infectivity) have often been reported in the literature, in vitro phage training has also been used to improve treatment with commercial or customized phage cocktails [179,180,181]. Phage adaptation was a common practice in the laboratories of the former Soviet Union in order to improve therapeutic cocktails, usually with the addition of a newly trained phage against the resistant pathogen [150]. Recently, in a randomized, placebo-controlled, and double-blind clinical trial investigating the effects of bacteriophages in UTI treatment, a commercially available cocktail (Pyo) was administered for 7 days. As is common practice, the Pyo bacteriophage cocktail was subjected to periodic adaptation cycles during the study [182]. To that end, Burrowes and co-workers described a simple method by which a phage cocktail, comprised of three phages, was evolved over 30 rounds of the Appelmans protocol on a suite of seven clinical and three laboratory strains of *P. aeruginosa* [172]. Only two of these bacterial isolates were sensitive to the original phages. The researchers showed that if each phage was allowed to evolve separately, the expansion of the host range was limited. On the contrary, when the three phages were evolved together, the progenies, after 30 rounds, were able to infect most or even all 10 bacterial strains. The genetic analysis revealed that this acquisition of broader host ranges was due to multiple recombination events between the phages of the cocktail. In an analogous manner, an improved cocktail of three phages against a panel of 110 *S. aureus* representative strains of clonal complexes in human infections was developed by mixing several wild-type phages and propagating cocktails on a subset of *S. aureus* strains [183]. The three final bred phages were found to have genomes intercrossed from up to three different ancestors as well as broader host ranges and increased virulence compared to them.

### 5.2. (B) Expanding Host Range by Phage Engineering and Reverse Genetics

Phage engineering has already yielded promising results as far as the artificial expansion of the phage host range is concerned, among the other benefits arising from the set of relevant technologies. In terms of host range, phage engineering aims to modify phages’ receptor-binding proteins (RBPs). RBPs, further divided into tail fibers and tail spike proteins, contain the domains responsible for recognizing the bacterial surface components [184].

Until now, researchers have employed various methods for the modification of these proteins. One approach known as “domain swapping” exchanges segments of RBPs, particularly in the C-terminal region of a phage with a narrow host range, with segments of the tail fiber’s C-terminal region of a heterologous phage with the desired host range [185]. There are different methods for this exchange to take place. For instance, Yao and colleagues employed the homologous recombination method, originally developed by Hamad et al. and subsequently modified by Yao et al. [186,187], to create a virulent, broad-host-range phage capable of infecting multiple *Burkholderia cepacia* complex (Bcc). Using this allelic replacement system, they replaced the C-terminal receptor-binding domain (RBD) of the Milagro (parental phage) tail fiber with that of the broad-host-range tailocin BceTMilo. First, they cloned a sequence encoding a region of the C-terminal end of the BceTMilo tail fiber and the full-length tail fiber assembly gene of BceTMilo in a carrier vector. Then, they subcloned this sequence into a suicide vector, introducing it into the *B. cenocepacia* AUA41545 (Milagro) lysogen. Inside the host, homologous recombination takes place between the Milagro lysogen and the plasmid construct, which enables domain swapping and the production of a recombinant phage with a broad host range [188]. Several groups followed the same approach for host range expansion [189,190]. Another strategy for modulating the host range is to introduce random or targeted sequence changes in the RBP genes through directed mutagenesis. This strategy is known as structure-directed mutagenesis [185]. For instance, Yehl and colleagues utilized a site-directed mutagenesis strategy, drawing inspiration from antibody specificity engineering, to generate functional variability within the host-range-determining regions (HRDRs) of the T3 phage tail fiber protein [191]. The researchers used that approach, thinking that the ability of these small regions in phage tail fibers to manipulate host specificity resembles the role of the three hypervariable regions of antibodies, known as complementarity-determining regions (CDRs), which define their specificity. As demonstrated in vitro and in a murine model, the resultant library of engineered phages, or “phage bodies”, included phages with altered host ranges, the ability to infect T3-resistant cells, and the suppression of bacterial phage resistance development [191].

Recently, the field of synthetic biology has presented revolutionary methodologies for the engineered expansion of host ranges. Levrier et al. developed Phage Engineering by In vitro Gene Expression and Selection (PHEIGES) using the T7 phage genome and *E. coli* cell-free transcription–translation (TXTL) [192]. According to the PHEIGES workflow, long PCR-generated fragments (<12 kbp) with overlapping sequences are re-assembled into T7 phage genomes in vitro using only an exonuclease. Batch TXTL reactions directly express the genomes, enabling the synthesis and selection of T7 phage variants with gene addition, deletion, or mutations. The authors assert that this methodology enables the production of 10^11^ pfu/mL of engineered phages in a single day. In addition, using PHEIGES, the researchers were able to expand the T7 phage host range as they created and selected variant T7 phages capable of infecting LPS-variant ReLPS *E. coli* strains that are not normally infected by T7 WT [192]. Other DNA assembly methods that have been reported recently, like SLiCE, Gibson and Golden Gate DNA assembly methods, as well as editing procedures based on CRISPR, may either not interface well with TXTL or may be more time-consuming and relatively costly and have been reviewed elsewhere [193,194,195]. In general, the methods followed in phage engineering may be divided into four categories, as follows: (i) genome editing and rebooting in native hosts, (ii) genome assembly in yeast and rebooting in native hosts, (iii) genome assembly in vitro and rebooting in native hosts or cross-genus hosts, and (iv) genome assembly in vitro and rebooting in a cell-free system [196]. Cheng et al., based on strategies (ii) and (iii), created the stepping-stone host-assisted phage engineering (SHARE) framework to promote virus synthesis in one pot. The researchers were able to successfully test the cross-genus and cross-order rebooting of 126 T7/non-T7-family phages that originally infect common clinical MDR strains, such as *K. pneumoniae*, *S. enterica*, *P. aeruginosa*, and *A. baumannii*, in stepping-stone hosts like *E. coli* DH10B, which is normally not permissive for any of these phages [196]. According to the authors, SHARE is an efficient and economic strategy for genome refactoring of viruses that can be applicable for phage engineering, targeting both pathogens and commensals.

## 6. Conclusions

On 21 November 2023, the World Health Organization (WHO) once again raised the alarm about the current situation with regard to the global rise in antibiotic resistance, which poses a significant health threat globally [197]. The 2022 Global Antimicrobial Resistance and Use Surveillance System (GLASS) report showed that in 76 countries, there is a significant concern regarding the resistance rates of certain pathogenic bacteria. Specifically, the report highlights a resistance rate of 42% for third-generation cephalosporin-resistant *E. coli*, 35% for methicillin-resistant *S. aureus*, and an alarming increase in the resistance of *K. pneumoniae* against critical antibiotics, including last-resort drugs such as carbapenems [198]. Moreover, the World Health Organization (WHO) has prioritized the research and development of novel vaccines, diagnostics, and medicines to address AMR in human health. One of the available strategies to deal with this threat is the recent revival of phage therapy. Although phage biology and its applications in the treatment of human diseases are linked to the origins of modern biology, surprisingly, only two clinical trials with an EudraCT protocol are listed on clinicaltrialsregister.eu (accessed on 8 May 2024) between 2014 and 2024 [199]. This simple fact highlights the challenges in successfully advancing phage therapy in a clinical setting. The European Medicines Agency (EMA) has recently acknowledged that certain principles from the “Guideline on the evaluation of medicinal products indicated for the treatment of bacterial infections” can be extended to the application of phages [200]. In the European Union (EU), due to the absence of a phage-based medicine license for human use, phage therapies are typically employed either as an independent experimental therapy or as a component of a clinical study. Considerable efforts have been made in this direction, with Poland and Belgium, among others, emerging as pioneers in the development of phage therapy [201,202]. In particular, Belgium applied phage treatments based on the magistral preparation of personalized phage products [202]. Phage therapy is also being explored as a potential conventional treatment for bacterial infections in France and Germany [203,204].

One of the key issues that has been extensively discussed by scientists and clinicians to make phage therapy a successful next-generation antimicrobial tool is the composition of therapeutic phage formulations for each clinical case, which is directly related to the host range [205]. Historically, researchers have tested all possible combinations [30,126,206]. Monophage therapy uses a single broad-range phage (Host Range Class I), typically derived from a phage bank, to apply a customized treatment known as “sur-mesure”. This kind of therapy has a narrow spectrum, has no significant impact on commensal bacteria, simplifies the steps before and after treatment, and allows for better control over the adverse effects of treatment, but it demands a large collection of phages and is prone to bacterial resistance. On the other hand, polyphage therapy has been applied either with a cocktail of Host Range Class I or even Class II phages. Polyphage therapy has been applied either with fixed (pret-a-porter) formulations or with a mix of fixed and adapted phages (through adaptive phage therapy). Polyphage therapy has a broad spectrum, is more likely to overcome bacterial resistance, and is better at resolving polymicrobial infections and biofilms, but it is more complex to propagate and test before and after treatment, may interfere with off-target microbiota, may cause adverse effects, may require regular updates against circulating strains, and makes final drug approval extremely complicated due to current regulations [143,207].

**Figure 2:**
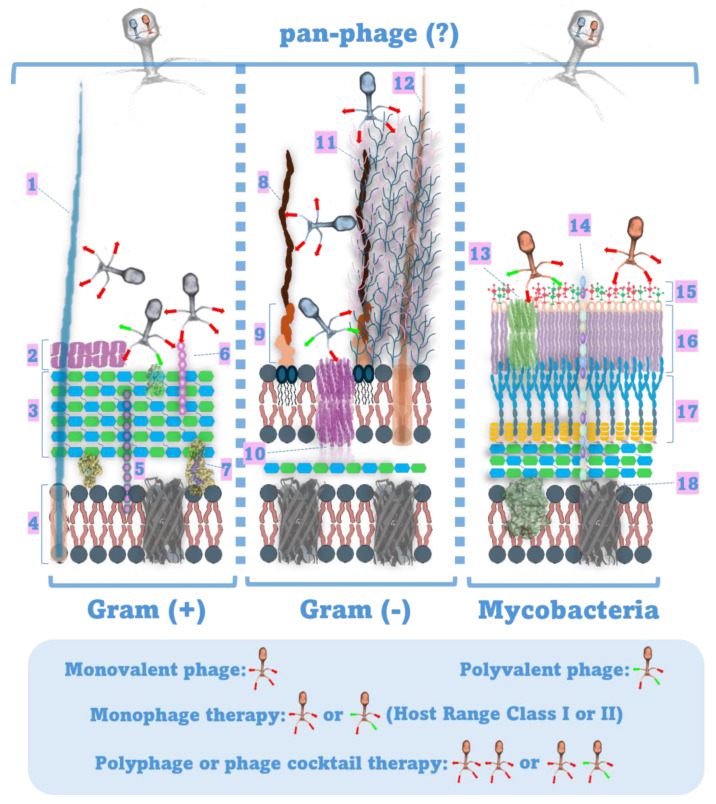
A pan-phage would be a phage that could cross the barriers of different types of bacterial cell walls. So far, no such phage has been found in nature or engineered in the laboratory. (1: flagella, 2: S-layer, 3: peptidoglycan layer, 4: cytoplasmic membrane, 5: lipoteichoic acid, 6: teichoic acid, 7: lipoprotein, 8: LPS O-antigen, 9: LPS core polysaccharide, 10: porin, 11: capsule, 12: pili, 13: porin, 14: lipoarabinomannan, 15: free glycolipids, 16: mycolic acids, 17: arabinogalactan layer, and 18: membrane protein. Detailed information about phage receptors on the bacterial surface is available inside the text.

The future of phage therapy seems to include all approaches. Customized treatments may provide better outcomes for patients, but they require well-organized centers that monitor local circulating strains, update their phage collections, and successfully adapt to the corresponding pathogen strains [208]. The ‘pre-a-porter’ approach, on the other hand, is required for the widespread application and establishment of phage therapy as an effective antimicrobial treatment. Fixed cocktails may be less effective for patients locally, but they are needed in areas where similar centers cannot be developed, have a known composition, and are therefore easier to approve for treatment. However, they must consist of a small number of broad-spectrum phages in order to be widely applicable [27]. In this direction, the discovery or creation of phages that can target specific pathogens belonging to both Gram-positive and Gram-negative bacteria would be important. As mentioned above, there are already traces of references to such phages or pan-phages (the word in Greek is “παν-φάγος” and literally means the organism that eats everything) in the literature. It is unclear whether such phages occur in nature as only a small percentage of environmental phages have been studied [209]. In addition, the increasing knowledge of the molecules that determine phage host range may allow us to engineer such broad-range phages in the future (Figure 2). Combined administration of antibiotics and phages could further enhance the action of such fixed cocktails. As has been shown in vitro and in vivo, phage–antibiotic synergy (PAS) can make phages more effective at controlling bacteria than when they are administered alone [210,211]. PAS has also been reported to reduce bacterial resistance not only to antibiotics but also to phages [171]. Of course, these and other observed benefits depend on the phage–host and phage–antibiotic combinations. These combinations need to be tested to eliminate negative effects that sometimes arise from this synergy, such as antagonism between phages and antibiotics [171]. In general, as we are all positively affected by the ease of use of antibiotics, we need beneficial solutions, such as phage therapy, that are versatile, easy to apply, and available to all patients to provide better treatment options at the clinical level.

## Figures and Tables

**Table 1:** Phages reported in the literature for the 2019–2024 period, categorized in a proposed classification system based on the taxonomic level of the hosts they infect.

A. Host Range to the Level of Species (Host Range Class I)					
Phage	Host	Host Range Results	Receptors	Family	Refs
vB_Sau_Clo6 and vB_Sau_CG	*Staphylococcus aureus* RN4220 strain	High killing activity (89% and 81%, respectively) against 44 isolated strains and 3 reference strains, including 11 MRSAs. The activity was comparable to that of the broad-range staphylophage K.	The bioinformatics analysis of the RBP genes provided no explanation.	*Myoviridae*	[91,92]
vB_SPuM_SP116	*Salmonella* Pullorum SPu-116	Broad lytic spectrum, infecting 27 out of 37 strains/several *Salmonella* strains, including 9 serotypes (Pullorum, Enteritidis, Indiana, Typhimurium, Infantis, Montevideo Heidelberg, Paratyphi A, Derby, and clinical isolates). However, there was no effect on other species of *Enterobacteriaceae.*	The authors speculated that the receptors of the SP116, like other *Salmonella* phages, including Felix O1, are located on the LPS, where different phages might bind with different moieties of the LPS.	*Myoviridae*	[93,94]
φMR003	*Staphylococcus* *aureus* RN4220 strain: Transformable strain, restriction-deficient (hsdR−), rsbU, agr-	Broad host range against 101 out of 104 (97%) MRSA clinical strains, broader than phages φSA012 (73%) and φΜR003 (57%).	Using bacterial deletion mutants and in silico analysis of φΜR003, the researchers suggested that the combined action of potential viral proteins contributes to a wide host range. Specifically, RBP belonging to the baseplate and tail proteins (ORF103) bind to the α-GlcNAc residues of RboP-WTA and to the WTA backbone (ORF105) of *S. aureus*, whereas the expression of a peptidoglycan hydrolase (ORF104) facilitates the infection.	*Herelleviridae* and *Silviavirus*	[95]
vB_EfaS_Hef13	12 *Enterococcus faecalis* strains	Broader host spectrum than previously isolated *E.faecalis* lytic phages. Successfully infected 12 out of 17 clinical strains of *E. faecalis* but not *E. faecium* reference strains.	Host range may be attributed to the presence of two ORFs (ORF55 and ORF75), which code for the receptor-binding protein in the phage tail apparatus, and a DNA methyltransferase, which protects phages from the bacterial restriction-modification system. On the other side of the host, a broader spectrum of HEf13 appeared to be associated with the potential receptor, the bacterial cell wall membrane protein, and the PIPEF since all *E. faecalis* strains that produced clear-plaques possessed the same amino acid sequence in the variable region of this receptor protein.	*Siphoviridae* and *Sap6virus*	[96]
Bp7 (T4-like phage)	*Escherichia coli*	Lytic activity against 16 out of 35 clinical strains of *E. coli* and 4 laboratory *E. coli* strains.	Host range was attributed to RBP gp38, which recognizes two OMPs (OmpC and LamB) as primary receptors and the heptose of the LPS core as a secondary receptor. RBP gp38 is located at the top of six long tail fibers (LTFs), recognizing suitable OMPs on the bacterial surface and binding to them reversibly, while small tail fibers (STFs) bind to the LPS irreversibly, allowing the phage to inject its genome into the host. So, the broad host range of Bp7 can be explained by the wide distribution of specific OMPs and the inner core of the LPS which is conserved among the several serotypes of *E. coli*.	*Myoviridae* (T4-like virus genus)	[97,98]
SHWT1	*Salmonella* Pullorum SP01	Lytic activity against nine *Salmonella* serovars, such as *Salmonella* Pullorum, *Salmonella* Gallinarum, *Salmonella* Enteritidis, *Salmonella* Typhimurium, *Salmonella* Derby, *Salmonella* London, *Salmonella* Typhi, *Salmonella* Heidelberg, and *Salmonella* Paratyphi B The phage was able to lyse intracellular Salmonella within macrophages and successfully protected mice against *Salmonella* Enteritidis and *Salmonella* Typhimurium infection.	No further information was available regarding the nature of the receptors involved.	*Siphoviridae*	[99]
vB_EcoM_KMB22 and vB_EcoM_KMB26	*Escherichia coli ST420* *Escherichia coli ST131*	Vkmb22 lysed 18 (44%) and Vkmb26 lysed 33 (82.5%) out of 40 local *E.coli* strains isolated from urinary tract infections. The undiluted cocktail was composed of vKMB22, and vKMB26 was able to lyse 33 strains (82%), while in the hundredfold diluted cocktail, spot lysis was observed in 23 strains (57%). The phage cocktail was species-specific and was not able to lyse strains of the *Enterobacter* and *Klebsiella* genera.	Both phages showed homology with the T4 genome. The main differences between phage genomes were observed in regions encoding for the long tail fibers, which are responsible for host specificity. In agreement with these variabilities, the phages had different host specificities. The broader host specificity of vKMB2 may be attributed to its isolation on the *E. coli* ST131 strain, which belongs to the predominant *E. coli* lineage among extraintestinal pathogenic *E. coli* isolates worldwide.	*Myoviridae*	[100]
ASEC2201 and ASEC2202	*Escherichia coli*	ASEC2201 formed lytic plaques in 40% of 50 clinical MDR *E. coli* isolates, while ASEC2202 formed lytic plaques in 44% of them. Both phages had survival percentages of 88% and 98% at a pH of 4, respectively, were able to grow at a low temperature, and were found to be stable in chloroform.	No further information was available regarding the nature of the receptors involved.	*Drexlerviridae*	[101]
**B. Host Range to the Intragenus Level (Host Range Class II)**					
**Phage**	**Host**	**Host Range Results**	**Receptors**	**Family**	**Refs**
ZoeJ (closely related to TM4)	*Mycobacterium smegmatis mc* *155*	Infected both fast- and slow-growing mycobacteria, including *M. tuberculosis* mc27000, *M. avium* Val14 (O), *M. bovis* BCG, and *M. interjectum* ATCC 51457, with a plating efficiency equivalent to that of *M. smegmatis*. No infection was observed on *M. avium* subsp. *silvaticum*, *M. abscessus* ATCC 19977, *M. simiae*, *M. avium* subsp. *avium* ATCC 25291, *Mycobacterium nonchromogenicum* ATCC 19530, or *Mycobacterium terrae* ATCC 15755, even when plated at high titer.	No further information was available regarding the nature of the receptors involved.	Cluster K	[102]
19 phages	Various strains of *Enterococcus*	Lytic activity against clinical isolates of *E. faecium* and *E. faecalis*, including both vancomycin-resistant *Enterococcus* (VRE) and vancomycin-susceptible *Enterococcus* (VSE). Eleven of 19 phages were able to lyse several strains, while three of them lysed almost all strains of *E. faecium* and *E. faecalis*	Under selective pressure, mutations primarily in the exopolysaccharide synthesis genes of *Enterococcus* strains were observed, suggesting that phage resistance may evolve by preventing phage recognition and initial binding.	10 *Siphoviridae* phages, 8 *Myoviridae* phages, and 1 *Podoviridae* phage	[103]
JC1 (Bcep22-like phage)	*Burkholderia cenocepacia* clinical isolate Van1	Impressively, it has a broad range against *Burkholderia* species, including *B. cepacia*, *B. multivorans*, *B. cenocepacia*, *B. stabilis*, *B. vietnamiensis*, *B. dolsa*, *B. ambifaria*, *B. anthina*, Bcc Group K, *Burkholderia* sp., and *Ralstonia pickettii*, which possesses high similarity to Bcc. There was lytic activity against 50 of the 85 strains, forming plaques on 29 of the 50 strains.	Using a collection of *B. cenocepasia* K56-2 LPS mutants, it was shown that the LPS inner core serves as the primary receptor.	*Podoviridae*	[104]
**C. Host Range to the Intergenus Level Within the Same Gram Category (Specificity Class III)**					
**Phage**	**Host**	**Host Range Results**	**Receptors**	**Family**	**Refs**
6 Atoyac phages	600 Gamma- proteobacteria retrieved from the same environment as isolated phages	Ιnfectious against bacteria from six different genera and three orders within the Gamma-proteobacteria class, namely, *Aeromonas*, *Pseudomonas*, *Yersinia*, *Hafnia*, *Escherichia*, and *Serratia.*	Although the comparative genome analysis identified the Atoyac phages as a novel viral group within the *Podoviridae* family, it could not provide more information about their remarkably broad host range spectrum.	*Podoviridae*	[105]
ΦΕent	*Salmonella enterica* serovars	Infected 11 of 22 tested *Salmonella* strains from nine different serovars, namely, Belem, Cerro, Enteritidis, Typhimurium, Kentucky, Infantis, Hadar, Thompson, and Braenerup), and three *Shigella* strains from two species (*S. dysenteriae* and *S. sonnei*).	No further information was available regarding the nature of the receptors involved.	*Siphoviridae*	[106]
10 T4-like phages	Six strains of prophage-free *Escherichia coli:* BL21, K12, EC101, DH5α, XL1 Blue, and Top10	Infected 61 out 72 strains of an *E. coli* collection and *E. coli* strain O157:H7 Δstx as well as the *S. sonnei* strain 53G.	The authors suggested that the observed cross-species infectivity of these T4-like phages could be attributed to the ability of T4 phages to bind to rough-type (R-type) LPS receptors, which are common in *Shigella* spp.	*Myoviridae*	[107]
EscoHU1	*Escherichia coli O157:H7 RIMD 0509939*	Able to form plaques not only in all *E. coli* O157:H7 strains tested but in strains belonging to other genera like *Citrobacter freundii* JCM 1657, *Salmonella bongori* CIP 82.33T, *S. sonnei* LMG 10473, and four serovars of *S. enterica* subsp. Enterica (Choleraesuis, Enteriditis, Infantis, and Typhimurium)	EscoHU1 binds to BtuB, which is a receptor that might contribute to its wide host range since the BtuB genes in *E. coli* and *Salmonella* are highly similar.	*Demerecviridae*	[108]
vB_YpeM_ MHS112 (MHS112) and vB_YpeM_GMS130 (GMS130	*Yersinia pestis*	Wide host range. Both phages infect the *Yersinia* genus, such as *Y. pseudotuberculosis* and *Y. enterocolitica*, as well as some species in the order of *Enterobacteriales*. More specifically, it infects *Shigella flexneri*, *E. coli* (ATCC 8739, ATCC 41446, and MG1655) and *Salmonella cholerasuis.* Furthermore, GMS130 was found to infect more non-*Yersinia* strains, including non-pathogenic *E. coli* (ATCC 25922 and FC 7792), enteroaggregative *E. coli* (EAEC), enterohemorrhagic *E. coli* (EHEC), *Shigella dysenteriae*, *Shigella boydii*, and *Enterobacter cloacae*, while the MHS112 phage was found to infect *Shigella flexneri*, *E. coli* (ATCC 8739, ATCC 41446, and MG1655), and *Salmonella cholerasuis*).	No further information was available regarding the nature of the receptors involved.	*Myoviridae*	[109]

## Data Availability

Not applicable.
